# The effectiveness of blended versus regular Forensic Outpatient Systemic Therapy in the treatment of juvenile antisocial behavior: a study protocol of a randomized controlled trial

**DOI:** 10.1186/s12888-023-04831-8

**Published:** 2023-05-04

**Authors:** S. Marjolein van Cappellen, Hanneke E. Creemers, Larissa Hoogsteder, Joan van Horn, Maja Dekovic, Jessica J. Asscher

**Affiliations:** 1 Department of Clinical Child & Family Studies, Utrecht University, P.O. Box 80140, Utrecht, 3584 CS Netherlands; 2 Faculty of Social and Behavioral Sciences, University of Amsterdam, P.O. Box 15776, 1011 NG Amsterdam, Netherlands; 3 De Waag, Outpatient forensic mental health care center, P.O. Box 1362, Utrecht, 3515 GA Netherlands

**Keywords:** Effectiveness, Randomized controlled trial, Forensic Outpatient Systemic Therapy (FAST), Antisocial behavior, Delinquency, Recidivism, Blended care

## Abstract

**Background:**

Antisocial behavior during adolescence can have long-lasting negative effects and leads to high societal costs. Forensic Outpatient Systemic Therapy (Forensische Ambulante Systeem Therapie; FAST) is a promising treatment for juveniles aged 12–21 showing severe antisocial behavior. The intensity, content and duration of FAST can be adjusted to the needs of the juvenile and their caregiver(s), which is considered crucial for effective treatment. Next to the regular version of FAST (FASTr), a blended version (FASTb) in which face-to-face contacts are replaced by minimally 50% online contacts over the duration of intervention was developed during the Covid-19 pandemic. The current study will investigate whether FASTb is equally effective as FASTr, and through which mechanisms of change, for whom, and under which conditions FASTr and FASTb work.

**Methods:**

A randomized controlled trial (RCT) will be carried out. Participants (*N* = 200) will be randomly assigned to FASTb (*n* = 100) or FASTr (*n* = 100). Data collection will consist of self-report questionnaires and case file analysis, and include a pre-test at the start of the intervention, a post-test immediately after the intervention, and a six month follow-up. Mechanisms of change will be investigated using monthly questionnaires of key variables during treatment. Official recidivism data will be collected at two-year follow-up.

**Discussion:**

This study aims to improve the effectiveness and quality of forensic mental health care for juveniles with antisocial behavior by studying the effectiveness of blended care, which has not been studied before in treatment of externalizing behavior. If found to be at least as effective as face-to-face treatment, blended treatment can help meet the urgent need for more flexible and efficient interventions in this field. In addition, the proposed study aims to unravel what works for whom, knowledge urgently needed in mental health care for juveniles with severe antisocial behavior.

**Trial registration:**

This trial was registered at ClinicalTrials.gov on 07/11/2022, registration number NCT05606978.

**Supplementary Information:**

The online version contains supplementary material available at 10.1186/s12888-023-04831-8.

## Background

Juvenile antisocial behavior, resulting in delinquent acts such as threatening, assault, property crime, and substance and weapon offences [1], can have long-lasting and devastating effects such as out of home-placement, recidivism, and delinquency during adulthood [2]. In addition, these juveniles have an increased risk of substance abuse and a criminal lifestyle during adulthood [3], and are less likely to have stable living situations, relationships, and work environments [4]. Juveniles with antisocial behavior negatively affect societal safety and induce high societal costs [5]. Considering the long-lasting personal and societal consequences of juvenile antisocial behavior [3–5], evidence-based treatment is vital for not only the juveniles and their systems, but also for society. However, juveniles with antisocial behavior are hard to reach and motivate for treatment. Prompted by the recent Covid-19 pandemic, the question rose whether interventions can be offered partially online, as this increases accessibility and potentially helps to involve these juveniles in treatment. The current protocol paper describes a randomized controlled trial (RCT) aiming to compare the effectiveness of blended versus regular Forensic Outpatient Systemic Therapy (Forensische Ambulante Systeem Therapie; FAST) [6], targeting severe antisocial behavior in juveniles.

### FAST

FAST is an outpatient systemic intervention for juveniles (aged 12–21 years) who show antisocial behavior and their multi problem families. The primary aims of FAST are to (1) reduce juvenile antisocial and/or delinquent behavior; (2) prevent out of home placement; and (3) prevent or decrease recidivism (risk) [6]. The secondary goals of FAST are to reduce substance use and contact with deviant peers, and to reach client formulated goals. FAST is based on the socio-ecological model by Bronfenbrenner [7] and addresses relevant systemic, family, and child factors using components that originate from cognitive behavioral therapy, system therapy, non-violent resistance and aggression regulation therapy.

FAST can be distinguished from other systemic interventions targeting antisocial behavior in juveniles by being especially adherent to the Risk Needs Responsivity (RNR) principles by Andrews and Bonta [8]. The RNR model is a leading and empirically well-substantiated model in criminology, and specifies that interventions should adhere to three principles in order to be effective: (1) the risk principle: non-intensive interventions should be offered to low recidivism risk clients, and high-intensive intervention should be offered to high recidivism risk clients; (2) the need principle: interventions should target the dynamic individual criminogenic needs during treatment; and (3) the responsivity principle: interventions should be responsive to the abilities of the client (system). FAST is very flexible in adhering to the RNR principles. FAST can be offered longer lasting and more intensive if needed, it addresses criminogenic risk and protective factors within the broad social context of a client system, and it is responsive to the abilities of the client system. Furthermore, FAST can be combined with other treatments to address the specific individual risk factors of a client (system). For instance, the intervention may combine individual therapy for caregivers with stress reduction or trauma therapy for juveniles.

FAST is a promising intervention targeting juveniles with antisocial behavior and their families [6]. Preliminary findings from pretest-posttest studies suggested that FAST resulted in some promising positive changes on the desired outcomes: FAST had a large effect in reducing general recidivism risk, a moderate effect in decreasing problems in the emotional/personal functioning of the juvenile, and a small to moderate effect in improving family functioning [9]. Additionally, FAST has been found to have sufficient program integrity [10], which is important as treatment integrity is generally associated with a higher treatment effectiveness [11]. However, more robust studies are needed to substantiate these results.

### Blended care: FAST blended (FASTb)

Prompted by the worldwide Covid-19 pandemic, a blended version of FAST has been developed (FASTb). Content wise, FASTb is nearly identical to regular FAST (FASTr). However, FASTb offers a combination of face-to-face and online treatment, consisting of a minimum average of 50% online direct treatment time over the duration of the intervention (such as phone calls, video calls, text messages, and eHealth; for more information, see Conditions). Blended interventions have several benefits over sole face-to-face intervention [12]. They increase accessibility and allow clients to work on therapy at any given moment, allow for precise registration of treatment delivery, increase (online) access to training materials for clients, involve lower time commitment for clinicians, and involve lower costs [13]. Moreover, blended interventions might be especially beneficial in the treatment of juveniles with antisocial behavior, as these interventions are expected to be even more flexible in adhering to the RNR principles than sole face-to-face treatment [14]. Blended intervention is less dependent on time and place, which increases the flexibility and accessibility of the intervention. It fits the increased use of the internet by juveniles and can be more responsive to the individual learning style or preference by offering both reading and visual material. Thereby, blended intervention might help reaching more clients and increase the involvement of this hard-to-reach target group in treatment. In fact, the integration of technological platforms in interventions has been employed to better reach juveniles and their families [15].

Despite these possible benefits, therapist implementation of blended interventions in forensic mental health care has been found to be disappointing, even though therapists viewed blended interventions as having potential to improve treatment quality [16]. The study of Kip et al. [17] investigated what could increase therapist use of blended interventions in forensic mental health care. Therapists indicated a need for more technological knowledge and highlighted the importance of the basic technological prerequisites, such as a stable internet connection. Further, they voiced that more research should be conducted to determine the actual effectiveness of blended interventions in the forensic field, and to specifically focus on why and for whom blended interventions work.

Unfortunately, to our knowledge, no research has investigated the effectiveness of blended interventions targeting juvenile externalizing behavior in general, let alone antisocial behavior or the complex and comorbid problems present in forensic youth care. Based on studies conducted on the effectiveness of blended intervention targeting several internalizing psychopathologies in juveniles, such as depression and anxiety, it can be concluded that blended treatment seems equally effective as face-to-face treatment. Previous research found blended interventions for depression and anxiety to be more effective than no intervention [18, 19] and active control groups [18], and equally effective as face-to-face interventions [20]. Further, a review showed that blended interventions aiming to reduce adult substance use were associated with lower dropout rates and greater abstinence than face-to-face interventions [12]. To determine if blended therapy is as effective as face-to-face therapy for juveniles with antisocial behavior, the current study is the first to investigate a blended intervention targeting juveniles with antisocial behavior by comparing the effectiveness of FASTb and FASTr.

### Mechanisms of change

According to the program theory [6], FAST aims to reach its primary and secondary goals by targeting risk factors at the level of the individual, the family, and the broader system of the juvenile. At the individual level, FAST targets criminogenic needs of the juvenile related to psychological functioning – such as cognitive distortions [21, 22]. At the family level, FAST is directed at improving caregiver-child relationship quality, caregiver behavior, and caregiver competence, and reducing conflicts between caregivers and juveniles [23, 24]. At the level of the broader system of the juvenile, FAST aims to target systemic risk factors by promoting social support [25], reducing interaction with deviant peers [26, 27], and decreasing truancy [28]. The current study will investigate whether the hypothesized mechanisms of change (social support, family functioning, and cognitive distortions) indeed contribute to the effectiveness of FASTb and FASTr.

### Moderators: what works for whom?

Intervention research continues to emphasize the importance of identifying subgroups for which and conditions under which interventions work best. Despite, there is still a lack of knowledge on what works for whom and under which conditions within interventions targeting antisocial and delinquent behavior of juveniles [29]. As such, the current study will investigate the influence of various moderators. Regarding treatment conditions, assessing program integrity (i.e., whether FAST is implemented as originally protocolled) is key, as non-significant or negative results may be caused by incorrect program implementation rather than an ineffective program [30]. Indeed, previous research has shown that treatment effectiveness in interventions targeting juvenile antisocial behavior is higher with higher treatment integrity [30–32], as well as stronger therapist-client alliance [33], higher treatment expectancies [34], and more social support [25]. Several studies have shown intervention effectiveness to also be higher with higher treatment cooperation [35], treatment motivation [36], and therapist experience [37], but these moderators have not yet been investigated for interventions targeting juvenile antisocial behavior. Further, demographic variables, such as gender [37], age, cultural background, and problem severity also influence treatment effectiveness [38]. By studying these moderating factors, we aim to determine which juveniles and families benefit most from FASTb and FASTr, and under which conditions.

### Aims of the study

The current study protocol describes an RCT comparing the effectiveness of FASTb and FASTr in the treatment of juveniles with antisocial behavior and their families. In addition, we will investigate the mechanisms of change of FASTb and FASTr, and for whom and under which conditions FASTb and FASTr work best. Thereby, new innovative steps will be taken to possibly improve the effectiveness and quality of mental health care for juveniles with antisocial behavior.

**Fig. 1:**
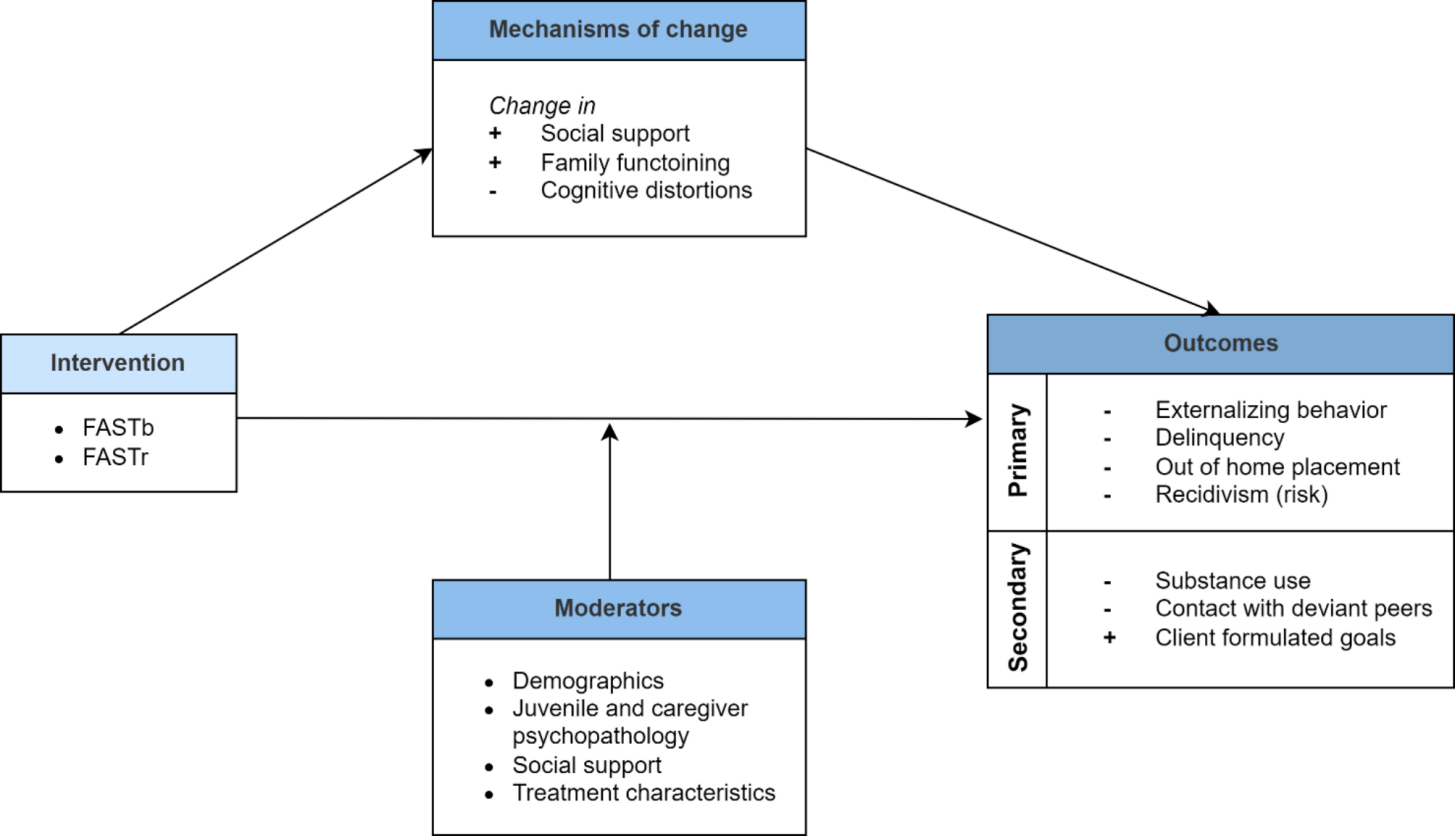
Conceptual model of study design

To this end, the aims of this study are threefold. The first aim is to compare the effectiveness of FASTb and FASTr in terms of reducing externalizing behavior, delinquency, out of home placement, and recidivism (risk) (primary treatment goals), and in terms of reducing substance use, contact with deviant peers, and in reaching client formulated goals (secondary treatment goals). It is expected that FASTb and FASTr are equally effective in reaching the primary and secondary goals of FAST. The second aim is to identify potential mechanisms of change in FASTb and FASTr. It will be investigated whether FASTb and FASTr, conform program theory, positively affect the intermediate outcomes: social support, family functioning (i.e., caregiver-juvenile conflict, caregiver-juvenile relationship quality, caregiver behavior, and caregiver competence), and cognitive distortions [6]. It is expected that these factors mediate the effectiveness of FASTb and FASTr. The third aim is to investigate what program and participant characteristics moderate FASTb and FASTr effectiveness. In other words, we aim to determine which families benefit most from FASTb and FASTr, and under which conditions. It is expected that the effectiveness of FASTb and FASTr is moderated by client factors (demographics such as gender and age, juvenile and caregiver psychopathology, and social support) and treatment characteristics (treatment integrity, treatment duration, intensity and completion, therapist-client alliance, motivation, expectancies, and cooperation). See Fig. 1 for a conceptual model of the research design.

## Methods

### Design

**Fig. 2:**
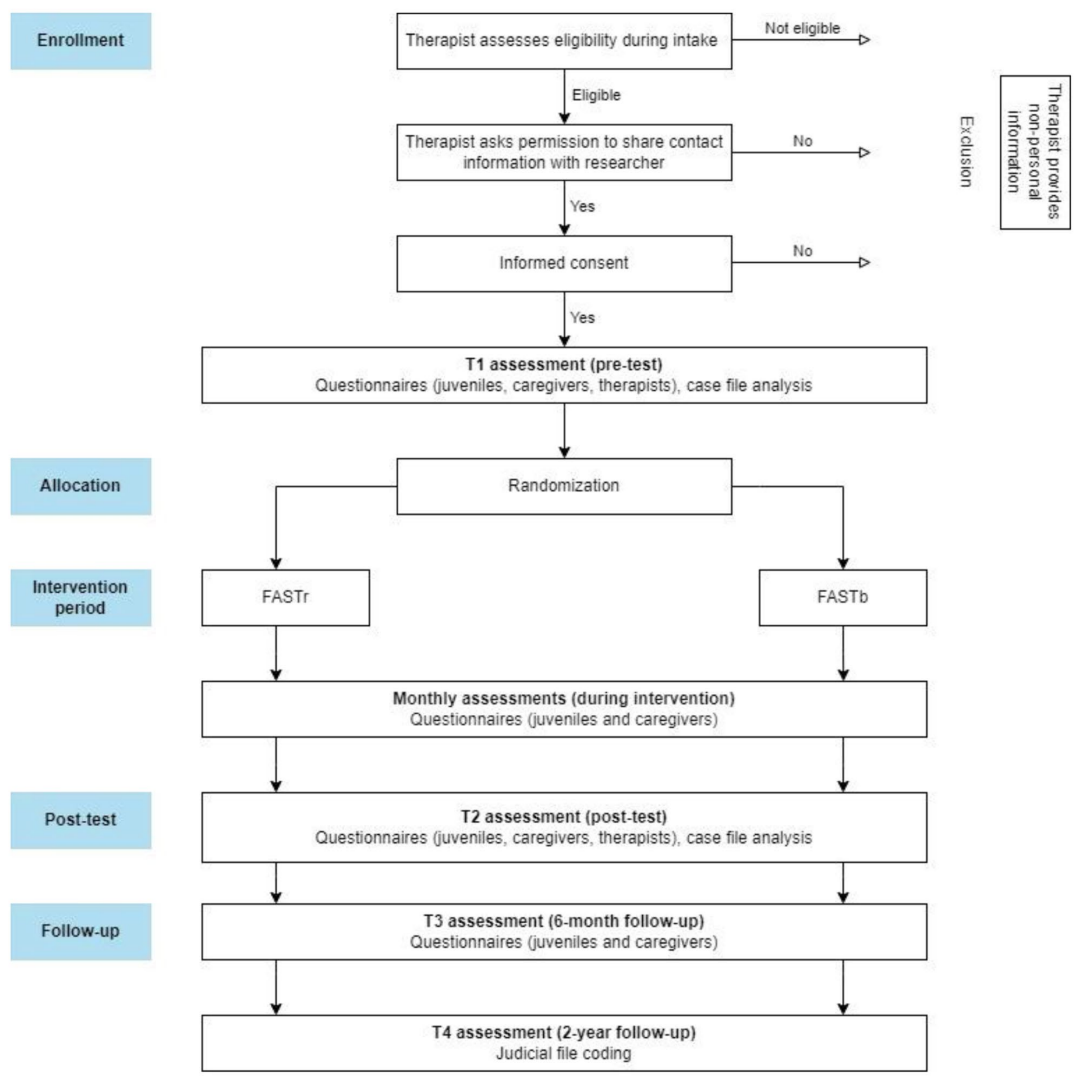
Flowchart of study design

This study is an RCT comparing two conditions: FAST-blended (FASTb; *n* = 100) and FAST-regular (FASTr; *n* = 100). The study has a multi-method (self-report, case-file analysis, and judicial file coding) and multi-informant (juveniles, caregivers, and therapists) design with four waves: pre-test, post-test, 6-month follow-up, and two-year follow up. To examine mechanisms of change in the total group (*n* = 200), monthly assessments of key variables are added during the treatment period. The study was registered at ClinicalTrials.gov on 07/11/2022 (NCT05606978). See Fig. 2 for the study flowchart and Table 1 for an overview of the various constructs, informants, and timing of assessments.

### Setting

FAST is offered by de Waag, an outpatient forensic mental health care center with 12 treatment sites in the Netherlands. Clients are referred by the juvenile justice system or voluntarily by mental healthcare facilities, school care coordinators, or general practitioners. FAST therapist teams at six treatments sites, located in Almere, Amersfoort, Amsterdam, Den Haag, Leiden, and Utrecht approach participants for the study. Participants will be recruited between 14 and 2022 and June 2025.

**Table 1:** Concepts, instrument, and informants at the different assessment points

Variable type	Instrument	Respondent					Assessment				
		Ju	Ca	Th	CF	JR	T1	M	T2	T3	T4
Primary outcome	YSR	x					x	x	x	x	
	CBCL		x				x	x	x	x	
	SDB	x					x	x	x	x	
	Out of home placement (case file)*				x		x		x		
	Out of home placement questionnaire	x	x						x	x	
	RAF GGZ Youth*				x		x		x		
	Convictions					x					x
Secondary outcome	Peilstation Middelengebruik	x					x		x	x	
	Substance use questionnaire	x						x			
	RAF GGZ Youth*				x		x		x		
	FAST Goal lists*				x		x		x		
	BPQ	x					x	x	x	x	
	FAST Goal lists*				x		x		x		
Mechanism of change	PSQ		x				x		x	x	
	NRI	x	x				x	x	x	x	
	IPPA	x					x	x	x	x	
	NPSI		x				x	x	x	x	
	NPQ	x	x				x	x	x	x	
	Parenting Practices	x	x				x	x	x	x	
	PDI	x	x				x	x	x	x	
	PCS-YR	x					x	x	x	x	
	BITI	x			x		x	x	x		
Moderator	Demographic questionnaire	x	x	x			x				
	ICU	x	x				x				
	APSD	x	x				x				
	Primary diagnosis (case file)				x		x				
	RAF GGZ Youth*				x		x				
	FAST evaluation form*				x				x		
	Treatment time registration				x				x		
	Direct treatment time		x					x			
	Relationship with Interventionist	x	x					x	x		
	TMS-F	x	x				x		x		
	PETS		x				x				
	Cooperation Scale	x	x	x					x		

*Note.* *Questionnaire is filled out as part of the standard FAST procedure. YSR = Youth Self Report; CBCL = Child Behavior Checklist; SDB = Self-report delinquent behavior; RAF GGZ Youth = Risk Assessment Instrument for Outpatient Forensic Mental Health Care Youth; FAST = Forensic Outpatient Systemic Therapy; BPQ = Basic Peer Questionnaire; PSQ = Parental Support Questionnaire; NRI = Network of Relationship Inventory; IPPA = Inventory of Parent and Peer Attachment; NPSI = Nijmeegse Parenting Stress Index; NPQ = Nijmeegse Parenting Questionnaire; PDI = Parenting Dimensions Inventory; PCS-YR = Psychological Control Scale Youth Report; BITI = Brief Irrational Thoughts Inventory; ICU = Inventory of Callous-Unemotional Traits; APSD = Antisocial Processes Screening Device; TMS-F = Treatment Motivation Scales for Forensic Outpatient Treatment; PETS = Parent Expectancies of Therapy Scale; Ju = Juvenile; Ca = Caregiver; Th = Therapist; CF = Case file analysis; JR = Judicial records; T1 = pre-test; M = monthly assessment; T2 = post-test; T3 = 6-month follow-up; T4 = 2-year follow-up

### Participants

In total, 200 FAST participants will be included in the study. The target group is diverse in terms of (comorbid) problems, but approximately 93% of the juveniles referred to FAST has a behavioral disorder [6] and they often grow up in families with multiple and complex problems. In FAST, 75% of juveniles is male.

#### Inclusion and exclusion criteria

Every juvenile and caregiver who receive FAST is considered for the study. FAST therapists determine whether clients meet inclusion and exclusion criteria during the standard FAST intake procedure. The inclusion criteria of FAST are: (1) Juvenile has an estimated IQ-score of 80 or higher and/or sufficient adaptive skills to benefit from the intervention; (2) Juvenile is aged 12–21 years old at intervention start; (3) Juvenile exhibits externalizing behavior that results in problems in at least two areas of life (family, school, leisure time), determined by clinical impressions based on referrer information and/or information from intake; (4) Juvenile has a medium to high recidivism risk, measured by the Risk Assessment Instrument for Outpatient Forensic Mental Health Care Youth (RAF GGZ Youth) [39]; (5) Presence of juvenile-caregiver relationship problems, as measured by the RAF GGZ Youth; (6) Juvenile has a diagnosis of a DSM-5 behavioral disorder, which is determined using case file analysis or a new diagnostic process; (7) Caregiver(s) and juvenile cannot be motivated to follow treatment at the treatment site after multiple attempts by the therapist; (8) Juvenile resides with their caregiver(s) or is expected to return to residing with caregiver(s) within the first two months of intervention.

The exclusion criteria of FAST are: (1) Juvenile exhibits severe psychiatric symptoms requiring admission; (2) Problem behavior of the juvenile is primarily caused by substance abuse problems and it is expected that treatment of the substance abuse problems will decrease the problem behavior; and (3) The safety of the therapist or family members cannot be guaranteed sufficiently. Additionally, clients that are not eligible to receive blended intervention are excluded from the study. These study exclusion criteria are: (1) Clients do not have an electronic device or suitable internet connection to receive blended care; (2) Clients have insufficient digital literacy to receive blended care; and (3) Families need a translator to receive the intervention.

#### Sample size calculation

Based on a MANOVA a priori power analysis, a group of *n* = 120 (*n* = 60 per group) will allow us to identify – with a power of 0.80, an alpha of 0.05 – small differences (d = 0.15) in effectiveness between FASTb and FASTr (G*power 3.1) [40]. To account for possible drop-out, which was approximately 20% in our previous comparable study [41], *n* = 200 participants will be recruited. Based on the number of juveniles referred to FAST at the treatment sites participating in this project (approximately 120 juveniles yearly), and participation rates of 70–90% in our earlier comparable studies [41, 42], it is considered feasible to include in total *n* = 200 juveniles and their caregivers within three years.

### Procedure

During the intake, therapists evaluate whether the clients meet the inclusion and exclusion criteria for the study. In case of doubt, therapists consult the research team in order to determine whether exclusion criteria are met. Once determined that a family can start FAST treatment, therapists ask caregivers and juveniles permission to share their contact details with the researcher. If they agree, FAST clients are approached by the main researcher or research assistants, whom all have signed a non-disclosure agreement and provided a certificate of conduct. Eligible clients receive verbal and written information about the study. The researcher obtains written informed consent from juveniles and caregivers for own participation, and from caregivers/legal representatives for juveniles younger than 16 years. After obtaining informed consent, participating families are randomized to receive FASTb or FASTr. Randomization is done on the level of the treatment site using a computerized randomization in a 1:1 ratio. Randomization outcome is shared by the researcher with both participants and participants’ therapist.

During the study, participants (juveniles and caregivers) fill out main questionnaires three times: Prior to or during the first weeks of intervention (pre-test; T1), immediately after the intervention (post-test; T2), and 6 months after termination of the intervention (6 months follow-up; T3). Therapists fill out questionnaires at T1 and T2. In addition, during the intervention, juveniles and caregivers fill out short monthly questionnaires. The number of monthly questionnaires depends on the length of intervention, which differs between five and nine months. Further, case file analysis is used to retrieve questionnaires that are filled out by juveniles, caregivers, and therapists as part of the standard FAST procedure. Official recidivism data will be collected at two-year follow-up (T4) to determine longer-term effects. For an overview of the study, see Fig. 2.

Given the complexity of the problems the target group faces, which often adversely affects the motivation to participate in treatment or research, researchers adjust the data collection to the preferences and agenda of the participants for timing and location (by (video) phone calls or at the homes of the families). Trained research assistants are available to assist with filling out the questionnaires, e.g., by taking them in interview form, and to carry out monthly assessments (online or by phone). Participants receive a financial compensation for filling out the questionnaires: 15 euros for T1, T2, and T3, and 1 euro per monthly assessment.

### Interventions

#### FAST

The treatment stage of FAST lasts five to nine months depending on the individual goals of the juvenile and the caregiver(s) and is followed by a period of aftercare (for more information on the treatment stages of FAST, see Table 2). At the start of treatment, therapists write an individualized basic Empirical Intervention cycle Summary (EIS). In the basic EIS, a problem analysis or function analysis of the problem behavior is described. The recidivism risk is determined and the safety for the juvenile, caregiver(s), and therapists and the degree of motivation are described. The basic EIS describes which FAST sub-goals need to be targeted to realize the main goal of FAST. During treatment, therapists evaluate the EIS every two weeks with the juvenile and the caregiver(s) and discuss which general and optional FAST sub-goals have the most priority. Interventions are selected based on the chosen sub-goals and by applying analysis circles. An analysis circle is created around a problem that is related to the chosen FAST sub-goal: On the right side of the circle, the influencers that contribute negatively to the problem behavior, or increase the problem behavior are described; on the left side of the circle, the influencers that reduce the problem behavior are described. Influencers can originate from various systems around the juvenile and family and are introduced by the juvenile and caregiver(s) themselves. When it is determined that the chosen sub-goals are reached, new goals are prioritized and new analysis circles are made. During treatment, the following supplementary modules can be selected for individual treatment: Stress and anger reduction, Impulse control, Self-control, Perceiving and interpreting correctly, Emotion-regulation, and Self-image. Every two months an evaluation takes place to determine whether longer treatment is needed with a maximum of nine months. In the last stage of the treatment, a future plan is developed that aims to prevent relapse.

**Table 2:** Treatment Stages of Forensic Outpatient Systemic Therapy (FAST) and Differences and Similarities Between FASTb and FASTr

Stage (duration)	Content	Differences and similarities between FASTb and FASTr
1. Preliminary referral (one month)	During this stage, all information needed to start a FAST trajectory is gathered by the professional. This phase consists of the registration, including viewing file information, contacting the referrer, and taking care of an intake interview. The intake interview takes place at the homes of the client and his/her caregiver(s) or at the treatment site of de Waag.	Intake is face-to-face in both conditions.
2. Pre-treatment (one month)	During this phase, making contact and motivation are central concepts. This phase is aimed at establishing contact, motivating, empowerment, making a safety plan and drawing up a treatment plan in the form of an Empirical Intervention Cycle Summary (EIS). In the EIS, a problem analysis is described, the recidivism risk is established and then monitored, as are motivation and safety for the juvenile, caregiver(s) and professionals. Every two weeks, the family discusses which general or optional sub-goals have the highest priority. At least three goals are worked on every two weeks. Preferably, attention is paid to realizing changes aimed at the family/ caregiver(s) in combination with changes aimed at the social domain (education and contact with friends) and individual domain (criminogenic needs) of the juvenile. When the sub-goals are met, new FAST goals are prioritized. In addition, professionals always pay attention to what is needed to guarantee or improve the safety of everyone and received support of the family members. An important goal is to improve the quality of contact and to reduce conflicts between caregiver(s) and the juvenile. Next, analysis circles are used to determine which interventions are most appropriate and will be implemented over the next two weeks.	First appointment after intake is face-to-face in both conditions.
3. Treatment (two to eight months)	In this stage, customized treatment is offered by working on standard FAST and optional FAST goals (addressed in supplementary modules). Regular FAST treatment consists of the Family-module, which first focusses on safety and connection, by using safety and crisis plans, and by learning to use a signaling plan. In addition, social support is organized via the network of the family. Second, parenting skills are improved. Selection of specific family modules is based on the needs of the caregiver. To do so, the EIS is evaluated and, if needed, changed every two weeks with the family and during the FAST intervision to determine the quality and to monitor the progress of the treatment. It is also assessed whether the interventions have actually led to change or whether they should be applied for a longer period of time	FASTr does not make use of eHealth or the GRIP-app. In FASTb, family modules are offered (partly) through Minddistrict.
4. Completion	The goals of the FAST treatment that were applicable to the juvenile and the family have been (largely) achieved. This will be confirmed by the presence of a future plan (focused on relapse prevention) and achieved results that are recognized by the FAST professional, the caregiver(s), referrer, and the juvenile.	The final session is face-to-face in both conditions.
5. Aftercare (one to three months, depending on risk level)	In the aftercare phase, the family works on their future plan with the support of significant others in their environment. In this phase, the FAST professional checks whether the juvenile and caregiver(s) are able to adhere to the plan for the future and whether the results can be maintained. Aftercare sessions will take place monthly.	Aftercare is completely online in FASTb, unless necessary to meet clients face-to-face.

Within FAST, treatment integrity is monitored closely. Every FAST therapist has succeeded the FAST basic training and offers FAST minimally 20 h per week. Each team has weekly FAST team meetings, during which treatments are monitored by evaluating the EIS’ and a bi-monthly treatment checklist, guided by an appointed therapist that is responsible for treatment integrity. At the end of the treatment, the FAST evaluation forms are completed by juveniles, caregivers, and therapists to verify compliance with the most essential FAST methods and techniques.

#### FASTr-condition

FASTr includes around 3 hours of face-to-face direct treatment time weekly. It consists of a maximum of 10% online direct treatment time, i.e., treatment via phone, video-calling or texting.

#### FASTb-condition

FASTb consists of a minimum average of 50% online direct treatment time over the duration of the intervention, such as phone calls, video calls, text messages, and eHealth. For more information on differences between FASTr and FASTb, see Table 2. eHealth involves the use of the software platform Minddistrict and the GRIP-app, a simple app which is combined with a heart rate watch on the client’s mobile telephone that registers physiological aspects of stress and anger and warns clients when they get aroused.

### Measures

An overview of the various constructs, informants and timing of assessments is presented below and provided in Table 1. Self-report and case file data will be collected. Case file analysis includes the retrieval of self-report questionnaires that are filled out by juveniles, caregivers and/or therapists as part of the standard FAST procedure, next to the coding of client information such as primary diagnosis (see below).

#### Primary outcomes

##### Externalizing behavior

Externalizing behavior will be measured at T1, T2, and T3 using juvenile self-report on the Externalizing scale of the Youth Self Report (YSR) [43] and caregiver report on the Child Behavior Checklist (CBCL) [44]. Both the YSR and CBCL contain 33 items, which are rated on a 3-point Likert scale ranging from 0 = *never* to 2 = *often*.

##### Delinquency

Delinquency will be measured at T1, T2, and T3 using juvenile self-report on the Self-report delinquent behavior (SDB) [45]. The SDB contains 30 items, asking the respondent to state the number of times they did certain things in the past year.

##### Out of home placement

Out of home placement will be coded from case files at T1 and T2 and will be measured at T2 and T3 by juvenile and caregiver report on a questionnaire measuring living situation. Four items assess where the juvenile lives most days of the week, which will be recoded into the categories 0 = *no* and 1 = *yes*.

##### Recidivism risk

Recidivism risk will be measured at T1 and T2 using the RAF GGZ Youth [39], which is filled out by therapists as part of the standard FAST procedure. Recidivism risk is rated on a 5-point Likert scale ranging from 1 = *low* to 5 = *high*.

##### Recidivism

Recidivism will be measured at T4 by coding if, when, and what type of crime juveniles were convicted for from official judicial records.

#### Secondary outcomes

##### Substance use

Substance use will be measured at T1, T2, and T3 using juvenile self-report questionnaire ‘Peilstation Middelengebruik’ [46] and case file analysis of the RAF GGZ Youth [46] and the FAST Goal lists. The Peilstation Middelengebruik contains five items measuring frequency and intensity of substance use (alcohol and drugs). The RAF GGZ Youth includes six items investigating the substance use of the juvenile. These items are summarized into a clinical judgment score ranging from 0 = *the client has no problem with alcohol and/or drug abuse now or in the past* to 5 = *the client currently has severe issues related to alcohol and/or drug abuse or dependence*. The FAST Goal lists are filled out by the juveniles, caregivers, and therapists, and contain one item investigating whether the juvenile uses substances (drugs or alcohol) and whether it leads to problems. The item is rated on a 10-point Likert scale ranging from 1 = *completely not true* to 10 = *completely true*.

##### Contact with deviant peers

Contact with deviant peers will be measured using juvenile self-report at T1, T2, and T3 on the Basic Peer Questionnaire (BPQ) [47]. The BPQ contains six items, which are rated on a 4-point Likert-scale ranging from 1 = *none* to 4 = *almost all of them*.

##### Client formulated goals

 Client formulated goals will be measured at T1 and T2 using case file analysis of the FAST Goal lists, which are filled out by juveniles, caregivers, and therapists as part of the standard FAST procedure. The FAST Goals lists consists of 21 items and provides a subjective measure of the effectiveness of FAST by asking respondents to rate whether they have reached the goals of FAST. The items are rated on a 10-point Likert scale ranging from 1 = *completely not true* to 10 = *completely true*.

#### Mechanisms of change

##### Social support

Social support will be measured at T1, T2, and T3 using caregiver self-report on the Parental Support Questionnaire (PSQ) [48]. The questionnaire contains 15 items, asking participants from whom or what they receive support (0 = *no* and 1 = *yes*) and, if they do, to rate their support satisfaction on a 5-point Likert scale ranging from 1 = *unsatisfied* to 5 = *satisfied*.

##### Family functioning

The following indicators of family functioning will be assessed: caregiver-juvenile conflict and relationship quality, caregiver behavior, and caregiver competence.

Caregiver-juvenile conflict will be measured at T1, T2, and T3 using juvenile and caregiver report on the Network of Relationship Inventory (NRI) [49]. The NRI contains six items, which are rated on a 5-point Likert scale ranging from 1 = *little to none* to 5 = *the most*.

Caregiver-juvenile relationship quality will be measured at T1, T2, and T3 using juvenile report on the Attachment scale of the Inventory of Parent and Peer Attachment (IPPA) [50, 51] and caregiver report on the Nijmeegse Parenting Stress Index (NPSI) [52]. The IPPA Attachment scale contains 12 items, which are rated on a 4-point Likert scale ranging from 1 = *almost never* to 4 = *almost always*. The NPSI Attachment scale contains 9 items, which are rated on a 6-point Likert scale ranging from 1 = *totally disagree* to 6 = *totally agree*.

Caregiver behavior will be measured at T1, T2, and T3 using juvenile and caregiver report on the Responsiveness and Consistency scales of the Nijmeegse Parenting Questionnaire (NPQ) [53], the Behavioral Control scale of the Parenting Practices questionnaire [54], three hypothetical situations from the Parenting Dimensions Inventory (PDI) [55] of which mean scores of two items measure Inductive Discipline, two items measure Harsh Discipline, and three items measure Other Punishments, and juvenile report on the Psychological Control Scale Youth Report (PCS-YR) [56]. The Responsiveness and Consistency scales of the NPQ contain 16 items, which are rated on a 6-point Likert scale ranging from 1 = *totally disagree* to 6 = *totally agree*. The Behavioral Control scale of the Parenting Practices contains six items, which are rated on a 5-point Likert scale ranging from 1 = *never* to 5 = *always*. The items on the PDI are rated on a 6-point Likert scale ranging from 1 = *very improbable* to 6 = *very probable*. The PCS-YR contains eight items, which are rated on a 6-point Likert scale ranging from 1 = *completely disagree* to 6 = *completely agree*.

Caregiver competence will be measured at T1, T2, and T3 using caregiver self-report on the Competence scale of the NPSI [60]. The scale contains 15 items, which are rated on a 6-point Likert scale ranging from 1 = *totally disagree* to 6 = *totally agree*.

##### Cognitive distortions

Cognitive distortions of the juvenile will be measured using case-file analysis at T1 and T2 of the Brief Irrational Thoughts Inventory (BITI) [57]. The BITI is filled out by juveniles as part of the standard FAST procedure and contains three subscales: Aggression and Justification (nine items), Sub-assertiveness (five items), and Distrust (four items). All items are rated on a 6-point Likert scale ranging from 1 = *totally disagree* to 6 = *totally agree*.

#### Moderators

##### Demographics

Participant demographics will be obtained at T1 using a questionnaire about demographic information. The demographics questionnaire contains nine items for juveniles, 19 items for caregivers, and eight items for therapists. The items measure gender, age, and ethnicity for all respondents. Additionally, for juveniles and caregivers, family composition is measured. For caregivers, work, and financial situation are measured. For therapists, educational degrees and work experience are measured.

##### Juvenile and caregiver psychopathology

Psychopathology of the juvenile will be measured at T1 using juvenile and caregiver report on the Inventory of Callous-Unemotional Traits (ICU) [58] and on the Narcissism (seven items) and Impulse control (five items) scales of the Antisocial Processes Screening Device (APSD) [59]. In addition, primary diagnoses of the juvenile are retrieved from case files. Psychopathology of the caregiver will be measured at T1 using case file analysis of the RAF GGZ Youth [39]. The ICU contains 24 items, which are rated on a 4-point Likert scale ranging from 0 = *not at all true* to 3 = *definitely true*. Items on the APSD are rated on a 3-point Likert scale ranging from 1 = *definitely not true* to 3 = *definitely true*. The RAF GGZ Youth contains one items asking therapists whether caregiver psychopathology is present. The item is rated on a 3-point Likert scale ranging from 0 = *not present* to 2 = *clearly present*.

##### Social support

See the description on Social Support in the section Mechanisms of Change.

##### Treatment characteristics

The following treatment characteristics will be assessed: treatment integrity, including adherence to the assigned level of online therapy, treatment duration, intensity and completion, therapist-client alliance, motivation, expectancies, and cooperation.

Treatment integrity will be measured at T2 using the FAST evaluation form, which is filled out separately by juveniles, caregivers, and therapists as part of the standard FAST procedure. The FAST evaluation form assesses whether the working elements of FAST were sufficiently applied during treatment (i.e., juveniles and caregivers report whether treatment goals were set in collaboration with them and therapists report whether they made sufficient use of cognitive behavioral therapy techniques) and whether treatment duration and appointment frequency matched the recidivism risk level. Treatment integrity will be scored in percentages, where a higher percentage indicates higher treatment integrity.

Adherence to the assigned level of online therapy within FASTb and FASTr will be measured at T2 by calculating the average percentage of online and face-to-face direct treatment time over the duration of intervention in two ways. First, it will be calculated based on registered direct time by therapists in their appointment agendas. Registration codes indicate whether the direct treatment time was online or face-to-face. In addition, caregivers will be asked monthly to report how many online appointments and how many face-to-face appointments they have had with their therapist in the past month. At T2, the average percentage of online and face-to-face appointments over the duration of the intervention will be calculated based on both measures.

Treatment duration, intensity, and completion will be assessed using case file analysis. Duration and intensity will be calculated based on the registered direct treatment time by therapists in their appointment agendas. Treatment duration will be measured in weeks and treatment intensity will be measured in average hours of direct treatment time per week. Treatment completion will be assessed by coding whether FAST completion was registered as positive or negative.

Therapist-client alliance will be measured at T2 using juvenile and caregiver report on the Relationship with Interventionist [60]. The Relationship with Interventionist contains 12 items, which are rated on a 6-point Likert scale ranging from 1 = *totally disagree* to 6 = *totally agree*.

Treatment motivation of juveniles and caregivers will be measured at T1 and T2 using self-report on the Treatment Motivation Scales for Forensic Outpatient Treatment (TMS-F) [61]. The TMS-F contains 16 items, which are rated on a 5-point Likert scale ranging from 1 = *totally disagree* to 5 = *totally agree*.

Treatment expectancies will be measured at T1 using caregiver report on the Parent Expectancies of Therapy Scale (PETS) [62, 63], and therapist report at T1 and T2 on one item asking how effective they think the assigned version of FAST (FASTb or FASTr) is in comparison to the other version of FAST. Therapists rate the question on a 5-point Likert scale ranging from 1 = *much less effective* to 5 = *much more effective*. The PETS contains seven items, which are rated on a 5-point Likert scale ranging from 1 = *low expectations* to 5 = *high expectations*.

Treatment cooperation will be measured at T2 using juvenile, caregiver, and therapist report on the Cooperation Scale [60]. The Cooperation Scale contains five items, which are rated on a 6-point Likert scale ranging from 1 = *totally disagree* to 6 = *totally agree*.

#### Monthly assessments

##### Juveniles

The monthly questionnaire for juveniles contains 34 items. It measures: (1) Externalizing behavior using five items of the Externalizing Scale of the YSR [43], of which some items are composites of multiple original items, by for example asking whether the juvenile stole something instead of differentiating between stealing inside and outside of the home; (2) Delinquency using six items of the SDB [45], of which some items are composites; (3) Substance use using six items, inquiring for both alcohol and drugs if the juvenile used the substance in the past week, on how many days, and whether the usage was representative for the past month; (4) Contact with deviant peers using two items of the BPQ [47], and one item asking whether the juvenile hang out with friends that fight; (5) Parenting using one item of the NPQ [53], one item of the IPPA [50, 51], two items of the NRI [49], and six items asking about monitoring, harsh punishments, and whether the juvenile got along with their caregiver; (6) Cognitive distortions using three items of the Aggression and Justification Scale of the BITI [57]; and (7) Treatment characteristics using one item of the Relationship with Interventionist Scale [60].

Items are measured using a 6-point Likert scale. Answer options are 1 = *never* to 6 = *often* for YSR and BPQ items, 1 = *0 times* to 6 = *more than 10 times* for SDB items, 1 = *little to none* to 6 = *the most* for NRI items, 1 = *almost never* to 6 = *almost always* for the IPPA item, and 1 = *totally disagree* to 6 = *totally agree* for other items.

##### Caregivers

 The monthly questionnaire for caregivers contains 16 items. It measures: (1) Externalizing behavior using three items of the CBCL [44], of which some are composites; (2) Parenting using three items of the NPSI [52], two items of the NRI [49], one item of the NPQ [53], and four items asking about monitoring, harsh punishment, and whether the caregiver got along with their child; and (3) Treatment characteristics using one item of the Relationship with Interventionist [60] and two items measuring direct treatment time (for more information, see the description in the section Treatment Characteristics).

Items are measured using a 6-point Likert scale. Answer options are 1 = *never* to 6 = *often* for CBCL items, 1 = *little to none* to 6 = *the most* for NRI items, and 1 = *totally disagree* to 6 = *totally agree* for other items.

### Data management

Contact data is stored in a digital double encrypted database. Research data is stored and will be analyzed in separate files, without direct links to the participants. Participants can fill out questionnaires on paper or online using personalized links send through Qualtrics, the online survey tool of Utrecht University. All completed paper documents are stored secured at Utrecht University, and will be scanned and directly stored on YODA, a research data management service that is compliant to the guidelines of General Data Protocol Regulation. Completed paper questionnaires will additionally be entered into Qualtrics by a researcher. Information from therapist files and judicial records will be coded into SPSS or JASP files. All data will be stored directly on YODA. Only the researchers involved in this study have access to the data.

### Plan of data analysis

Data will be analyzed according to the intention-to-treat principle and will also be analyzed separately for the completers only. Little’s test will be used to test whether data is missing at random. If so, missing data will be imputed so that all participants can be included in the analyses. Possible baseline differences in demographic characteristics between the participating and non-participating FAST-clients will be checked by means of chi-square analyses and independent t-tests to investigate the representativity of the included sample. In addition, possible baseline differences in demographic characteristics and outcome variables between the FASTb and FASTr condition will be checked by means of (M)ANOVA (continuous variables) and chi-square analyses (categorical variables). If (some of) these variables show significant differences between FASTb and FASTr conditions, they will be entered as covariates in all (regression) models testing the effectiveness of the intervention.

#### Effectiveness

Research questions related to the effectiveness of FASTb versus FASTr on the primary and secondary outcomes will be answered using co-variance analyses with a correction for multiple testing, with the pre-test (T1) score on the dependent variable as a covariate, the post-test (T2) and 6-month follow-up (T3) scores as the dependent variable, and the condition (FASTb or FASTr) as a factor. Dichotomous outcome variables will be tested with Chi-square analysis to compare the percentage of the FASTb and FASTr conditions. Recidivism data after two years (T4) will be analyzed using Kaplan Meyer and Cox survival analyses. Effect sizes will be computed as Cohen’s d, based on adjusted means and standard errors.

#### Mechanisms of change

The research questions related to the expected mechanisms of change (social support, family functioning, and cognitive distortions) will be investigated by testing whether changes in the hypothesized mechanism of change predict change in the primary and secondary outcomes.

#### Moderation

The research questions related to the moderators (demographics, juvenile and caregiver psychopathology, social support, and treatment characteristics) will be answered by including an interaction term (moderator*condition) to the model with a correction for multiple testing. Post-hoc analyses for moderator effects will be conducted by splitting the file according to the moderator and again conducting an ANCOVA and calculating effect sizes separately for each group. Regression analyses will be conducted for the continuous moderators, and multi-group analyses will be performed for dichotomous variables.

## Discussion

This study protocol describes an RCT comparing the effectiveness of FASTb and FASTr in the treatment of juveniles with antisocial behavior and their families. In addition, we will investigate the mechanisms of change of FASTb and FASTr, and for whom and under which conditions FASTb and FASTr work best. The hypotheses are that (1) FASTb and FASTr are equally effective in reaching the primary and secondary goals of FAST; (2) conform program theory, social support, family functioning, and juvenile cognitive distortions mediate effectiveness of FASTb and FASTr; and (3) the effectiveness of FASTb and FASTr is moderated by demographics, juvenile and caregiver psychopathology, social support, and treatment characteristics.

In the past years, blended mental health care has increased due to the need for time-efficient and cost-effective interventions, the development of digital tools, and the further digitization of society [64]. Blended treatment has several benefits over sole face-to-face therapy such as increased accessibility, lower time commitment for clinicians, and lower costs [12]. Moreover, for juveniles with antisocial behavior, blended treatment might be even more beneficial as it lends itself to be even more flexible in adhering to the RNR principles than sole face-to-face treatment. Although blended treatment has been shown to be effective in several studies investigating internalizing problems [12, 18, 20], this study will be the first to investigate the effectiveness of blended treatment for juveniles with antisocial behavior. Thereby, this study aims to improve the effectiveness and quality of forensic mental health care for these juveniles.

In the recruitment and data-collection of this study, several challenges are expected. One will be the recruitment and retainment of *n* = 200 FAST clients, as the target group is generally hard to reach and motivate for research. We have planned several actions to promote participation and retainment. First, we include six treatment sites to be able to approach sufficient clients. If influx of FAST clients or willingness of FAST clients to participate in the study are lower than expected, other treatment sites can participate as well. To ensure an efficient participation of a new treatment site, all treatment sites of de Waag that offer FAST have been informed of the study, and the responsible FAST therapists of all treatment sites have been informed about the study procedure. Second, we will invest time and effort in the recruitment and retention of participants. The research team will adjust the recruitment and data collection to the preferences of the participants for timing and location. For instance, questionnaires can be filled out on paper or online, or can be taken in interview form by trained research assistants during (video) calls or home visits. Third, participants will receive a financial compensation for each completed measurement. When completing all measurements, each participant will receive around 50 euros. We have good experiences in increasing motivation to participate by paying participants for their participation [41, 42]. Fourth, twice a year, we will organize meetings with our advisory council, which consists of professionals and former FAST-clients, to keep reflecting on our study procedure and gain new ideas on how to promote study participation and retention.

A second challenge of the study can be therapist adherence to the set percentages of direct online treatment time for FASTb and FASTr. Percentages of direct online treatment time are not calculated automatically within the appointment registration system. Therefore, percentages will be calculated every three months. In addition, the researcher and therapists are in close contact and meet frequently to discuss potential issues regarding adherence.

Despite the potential challenges, the current study has several important strengths. First, the study involves a rigorous design. We will conduct an RCT, which is the golden standard of intervention evaluation [65]. By investigating both short-term and long-term effectiveness of FASTb and FASTr, we will be able to detect possible sleeper effects after six months and two years. Second, the multi-informant (i.e., juveniles, caregivers, and therapists) and multi-method (i.e., self-report, case-file analysis, and judicial file coding) design of the study has several strengths. Self-report of delinquent behavior is known to be systematically under- and over-reported [66], and actual convictions pose a more objective measure of delinquency. However, actual convictions are not representative of all delinquent behavior, and self-report might detect delinquency that might not have been detected or convicted by justice. Further, the measurement of actual convictions makes our study results most relevant to authorities, as it is consistent with government practices in the operationalization of recidivism (i.e., actual convictions) [67]. Third, the study was designed in cooperation with both clients and therapists, which strengthens the study in its practical feasibility and increases therapist motivation for participation in the study. Fourth, by minimizing exclusion criteria for this study, we have maximized the chances to include a representative sample. As a result, the study can provide information on a generally understudied and hard-to-reach group.

Our study will have several important clinical implications. This study will improve our knowledge on the potential benefits of blended care for juveniles with severe antisocial behavior, thereby possibly improving the effectiveness and quality of forensic youth care. By investigating mechanisms of change, we will be able to inform clinical practice on which mechanisms of change contribute more and less to the effectiveness of FASTb and FASTr. Thereby, therapists could accentuate the working mechanisms during intervention to reach optimal effectiveness and motivate towards clients why certain aspects are worth investing in during intervention. By investigating moderators, our study will be able to inform clinical practice for whom and under what circumstances FASTb and FASTr are most effective. Thereby, targeted implementation of FASTb and/or FASTr can be substantiated. Furthermore, the study will provide important scientific knowledge on what works in involving and treating this hard-to-reach clinical group. In short, the results of our study will contribute to the justification of the funding, efforts, and time investment that therapists, families, and policy makers dedicate to FAST. Potentially, more effective, tailored, accessible, and efficient treatment can be offered to juveniles with antisocial behavior and their families.

## Conclusion

The present study aims to compare the effectiveness, mechanisms of change, and moderators of FASTb and FASTr. Evidence-based treatment is vital for not only juveniles with antisocial behavior and their systems, but also for society. If found to be at least as effective as face-to-face treatment, blended treatment can help meet the urgent need for more flexible and efficient interventions in juvenile justice context. The results of this study are of importance to all countries that aim to treat juveniles with antisocial behavior effectively and efficiently.

## Electronic supplementary material

Below is the link to the electronic supplementary material.

Supplementary Material 1 SPIRIT Checklist

## Data Availability

Due to the sensitive nature of our data, the data will not be publicly available. However, other researchers can request access to (part of) the data by sending a well-substantiated proposal after research questions have been answered to the corresponding author (Marjolein van Cappellen). The research team will evaluate proposals and decide whether and which parts of the data will be shared. Data will always be shared anonymized and (part of) the research team will be involved with the proposed study.

## Abbreviations

FAST(r): Forensic Outpatient Systemic Therapy
FASTb: Blended Forensic Outpatient Systemic Therapy
RCT: Randomized Controlled Trial
RNR: Risk Needs Responsivity
RAF GGZ Youth: Risk Assessment Instrument for Outpatient Forensic Mental Health Care Youth
EIS: Empirical Intervention cycle Summary
YSR: Youth Self Report
CBCL: Child Behavior Checklist
SDB: Self-report delinquent behavior
BPQ: Basic Peer Questionnaire
PSQ: Parental Support Questionnaire
NRI: Network of Relationship Inventory
IPPA: Inventory of Parent and Peer Attachment
NPSI: Nijmeegse Parenting Stress Index
NPQ: Nijmeegse Parenting Questionnaire
PDI: Parenting Dimensions Inventory
PCS-YR: Psychological Control Scale Youth Report
BITI: Brief Irrational Thoughts Inventory
ICU: Inventory of Callous-Unemotional Traits
APSD: Antisocial Processes Screening Device
TMS-F: Treatment Motivation Scales for Forensic Outpatient Treatment
PETS: Parent Expectancies of Therapy Scale
(M)ANOVA: (Multivariate) analysis of variance
ANCOVA: Analysis of covariance
